# miR-1180–3p as a biomarker of obstructive sleep apnea hypopnea syndrome and its role in chronic intermittent hypoxia-induced vascular injury

**DOI:** 10.1016/j.clinsp.2026.100944

**Published:** 2026-04-23

**Authors:** Mulan Deng, Yanjie Chen, Hongye Chen, Qihui Chen

**Affiliations:** Pulmonary and Critical Care Medicine, Fuzhou Hospital of Traditional Chinese Medicine Affiliated to Fujian University of Traditional Chinese Medicine, China

**Keywords:** miR-1180–3p, Obstructive Sleep Apnea Hypopnea Syndrome, Chronic Intermittent Hypoxia, Oxidative Stress, CERS1

## Abstract

**Background:**

Obstructive Sleep Apnea Hypopnea Syndrome (OSAHS), a common sleep-related breathing disorder, is closely associated with an increased risk of cardiovascular and metabolic comorbidities. Polysomnography, the diagnostic gold standard, is costly and environmentally sensitive. This study explored the diagnostic potential of miR-1180–3p and its role in Chronic Intermittent Hypoxia (CIH)-induced vascular injury.

**Methods:**

Serum miR-1180–3p and CERS1 levels were measured in 112 OSAHS patients and 88 controls via qRT-PCR. Correlations with PSG parameters (LSpO₂, AHI, TS90%) were analyzed. In vitro, HUVECs were exposed to CIH, and miR-1180–3p effects on cell viability, apoptosis, and oxidative stress (MDA, SOD, GSH-Px) were assessed. TargetScan and dual-luciferase assays validated miR-1180–3p-CERS1 binding. Evaluating miR-1180–3p/CERS1 axis regulation of OSAHS pathogenesis via co-inhibition experiments.

**Results:**

A significant upregulation of miR-1180–3p was detected in OSAHS patients (p < 0.001). miR-1180–3p served as an independent risk factor for OSAHS (OR = 5.405, 95% CI 2.247‒13.001) and held diagnostic value (AUC = 0.882). miR-1180–3p correlated negatively with LSpO_2_ (*r* = -0.705) but positively with AHI (*r* = 0.676) and TS90% (*r* = 0.774), with correlations persisting across mild, moderate, and severe OSAHS subgroups. In CIH-treated HUVECs, miR-1180–3p inhibition restored cell viability, reduced apoptosis, and alleviated oxidative stress. CERS1 was confirmed as a direct target, and its silencing reversed miR-1180–3p inhibition-mediated protection.

**Conclusion:**

miR-1180–3p was a novel diagnostic biomarker for OSAHS. By targeting CERS1, miR-1180–3p exacerbated CIH-induced damage to HUVECs, thereby regulating the pathological progression of OSAHS.

## Highlights

• miR-1180–3p is significantly overexpressed in the serum of patients with OSAHS.
• miR-1180–3p acts as a potential diagnostic biomarker for OSAHS.
• miR-1180–3p is an independent risk factor for the occurrence of OSAHS.
• Downregulation of miR-1180–3p alleviates CIH-induced injury in HUVECs.
• miR-1180–3p targets CERS1 to regulate CIH-induced injury in HUVECs.

## Introduction

Obstructive Sleep Apnea-Hypopnea Syndrome (OSAHS) is a common sleep-disordered breathing disorder strongly associated with cardiovascular and metabolic comorbidities.^1^ Currently, the diagnosis of OSAHS centers on polysomnography (PSG), which can accurately record multiple parameters, including respiration and blood oxygen levels.^2^ However, its application remains constrained by several limitations: PSG devices are costly and require professional operation,^3^ and some patients may experience disrupted sleep due to environmental interference, leading to significant fluctuations in data reliability.^4^ Therefore, identifying reliable biomarkers for the early identification, condition assessment, and efficacy monitoring of OSAHS holds substantial clinical value and public health significance.

MicroRNAs (miRNAs) are key post-transcriptional regulators of gene expression and have been implicated in OSAHS pathogenesis.^5^ For instance, miR-149–3p has been detected to be significantly upregulated in the serum of OSAHS patients and may have potential diagnostic value for OSAH;S^6^ miR-126 regulates OSAHS progression by targeting HIF-1α.^7^ Notably, miR-1180–3p was identified as significantly upregulated in OSAHS patients in a serum miRNA profiling study.^8^ Although the roles of many miRNAs in the development of OSAHS have been clarified, the specific functions of many miRNAs, including miR-1180–3p, remain less studied. This miRNA is of particular interest given its established relevance in vascular biology and cardiovascular disease. Currently, miR-1180–3p has been validated by numerous studies as a biomarker with significant potential value in the pathological processes of diverse diseases, including colorectal cancer^9^ and hepatocellular carcinoma.^10^ It regulates apoptosis and proliferation in vascular contexts,^11^ and is associated with platelet activity and recurrent cardiovascular events in coronary artery disease.^12^ Since endothelial dysfunction is a critical link between OSAHS and elevated cardiovascular risk,^1^ based on the above research background, it is preliminarily speculated that miR-1180–3p may exert a regulatory effect on the disease progression of OSAHS. However, its specific role and diagnostic value in OSAHS remain unexplored.

Ceramide Synthase-1 (CERS1) is a key enzyme in ceramide biosynthesis.^13^ Ceramide can alter mitochondrial outer membrane permeability and ultimately lead to apoptosis in tumor cells.^14^ As a key enzyme in ceramide synthesis, CERS1 plays a critical regulatory role in this apoptotic process. A study by Xu Y et al. found that CERS1 is downregulated in patients with non-small cell lung cancer and exerts anti-tumor effects by downregulating the PI3K/AKT/mTOR signaling pathway.^15^ Additionally, many stress stimuli, including oxidative stress inducers and anti-cancer drugs, can promote the generation of endogenous ceramide by activating CERS1, thereby inducing ceramide stress and triggering cell death.^16^ Therefore, the authors hypothesize that CERS1 may be linked to OSAHS; however, current research on its role in OSAHS remains limited.

This study investigates the diagnostic efficacy of miR-1180–3p in OSAHS and evaluates its role in disease progression using a Chronic Intermittent Hypoxia (CIH) model and elucidates whether its mechanism involves direct regulation of CERS1. The ultimate goal is to explore new directions for the early diagnosis and targeted therapy of OSAHS.

## Patients and methods

### Study subject

The reporting of this study adheres to the Strengthening the Reporting of Observational Studies in Epidemiology (STROBE) statement.

In total, 112 subjects with OSAHS who presented to Fuzhou Hospital of Traditional Chinese Medicine Affiliated to Fujian University of Traditional Chinese Medicine were enrolled in this study, and 88 healthy individuals who received physical examinations at the same outpatient clinic over the corresponding period were included as the control group. Ethical approval for this study was granted by the Ethics Committee of Fuzhou Hospital of Traditional Chinese Medicine Affiliated to Fujian University of Traditional Chinese Medicine (n°2,021,126, on 11 September 2021).

Inclusion Criteria: 1) All patients underwent nocturnal PSG examination. 2) All patients fulfilled the diagnostic criteria for OSAHS. 3) All patients had complete clinical data. 4) An informed consent form was voluntarily signed by all patients.

Exclusion Criteria: 1) Patients diagnosed with other sleep disorder conditions. 2) Patients with other respiratory diseases. 3) Patients with malignant tumors. 4) Pregnant or lactating women.

Levels of Total Cholesterol (TC), Triglyceride (TG), High-Density Lipoprotein Cholesterol (HDL-C), and Low-Density Lipoprotein Cholesterol (LDL-C) were analyzed in all enrolled subjects using an automatic biochemical analyzer. General information, such as age, gender, Body Mass Index (BMI), and history of hypertension or diabetes, was also collected. PSG was performed to monitor sleep parameters, including the Lowest Pulse Oxygen Saturation (LSpO_2_), Apnea-Hypopnea Index (AHI), and percentage of sleep time with oxygen saturation < 90% (TS90%). The PSG results were analyzed and interpreted by a qualified physician. According to standard guidelines, patients were stratified into mild (5 < AHI ≤ 15), moderate (15 < AHI ≤ 30), and severe OSAHS (AHI > 30) groups.

### Sample collection

On the first morning of hospitalization, all OSAHS patients provided 10 mL of fasting venous blood, while the same amount of fasting venous blood was drawn from control individuals during their morning physical examination. All blood samples were centrifuged at 3500 rpm for 20 min at 4 °C, and the supernatants were collected and stored at −80 °C for subsequent experiments.

### Quantitative reverse transcription polymerase chain reaction (qRT-PCR)

Total RNA was extracted from samples using TRIzol reagent (Invitrogen, USA). cDNA synthesis was performed following the manufacturer’s instructions of the PrimeScript RT kit (TaKaRa, Japan). qRT-PCR detection was conducted using a 7500 Real-Time PCR System (Applied Biosystems, USA). U6 was employed as the reference gene, and the relative expression levels were determined via the 2^−ΔΔCt^ method. The PCR program was set as follows: 95 °C pre-denaturation for 30 s; 95 °C for 5 s, 60 °C for 30 s, for 40 cycles. The primer sequences are listed as follows: miR-1180–3p, forward 5′-CAGAAACAGCCATCCCAGAG-3′, reverse 5′-GCCTTCAGCAGGATGTCAAT-3′; CERS1, forward 5′-CCTCCAGCCCAGAGAT-3′, reverse 5′-AGAAGGGGTAGTCGGTG-3′; U6, forward 5′-CTCGCTTCGGCAGCACATAT-3′, reverse 5′-ACGCTTCACGAATTTGCGTC-3′.

The primer sequences for CERS1 were validated using NCBI Primer-BLAST to ensure specificity. Although their length design is atypical, they were evaluated by the synthesis supplier (GenePharma, Shanghai) based on thermodynamic parameters and confirmed to be suitable for subsequent qRT-PCR experiments.

### Cell culture

Endothelial cells obtained from the Human Umbilical Vein (HUVECs) were purchased from ATCC (USA). Cells were seeded in Dulbecco’s Modified Eagle Medium (DMEM) containing 10% Fetal Bovine Serum (FBS) and 1% penicillin-streptomycin, and then cultured in a 37 °C, 5% CO₂ incubator.

For Chronic Intermittent Hypoxia (CIH) treatment, HUVECs were exposed to a cyclic hypoxic protocol: each cycle consisted of 15 min under normoxic conditions (21% O_2_, 5% CO_2_, 74% N_2_) followed by 15 min under hypoxic conditions (5% O_2_, 5% CO_2_, 90% N_2_). This cycle was repeated until the total treatment duration reached 24 h, with a total of 48 cycles. All procedures were performed in a Heracell™ Vios 250i CO_2_ incubator (Thermo, 51,033,605, USA). Control cells were maintained under normoxic conditions without CIH exposure.

### Cell transfection

miR-1180–3p mimics, miR-1180–3p inhibitors, si-CERS1, and their corresponding negative controls were designed and synthesized by GenePharma (Shanghai). Logarithmically growing HUVECs were collected and seeded at a density of 5 × 10⁴ cells per well, then the cells were plated in 6-well plates. Plasmid transfection was performed following the instructions of Lipofectamine™ 3000 transfection reagent (Invitrogen, L3000150, USA). Briefly, 10 μL of transfection reagent and 10 μL of plasmid were each diluted into 500 μL of serum- and antibiotic-free culture medium. The diluted reagents were mixed thoroughly and then incubated at room temperature for 20 min. Additional serum-free and antibiotic-free culture medium was then combined with the mixture, and the cells were incubated in a 37 °C, 5% CO_2_ incubator for 4-hours. Afterward, the transfection medium was discarded and replaced with complete medium containing 5% FBS, and the cells were further cultured for 24 h.

### Cell counting kit-8 (CCK-8) assay

Cell viability was measured using the CCK-8 assay. HUVECs were seeded into 96-well plates at a density of 5 × 10³ cells per well and cultured in an incubator until cell adhesion was achieved. Subsequently, 10 μL of CCK-8 reagent was added to each well, and the plates were incubated at 37 °C in a cell incubator with light shielding for 4 h. The absorbance at 450 nm was then measured using a microplate reader (Thermo Scientific, USA).

### Flow cytometry

HUVECs cells were collected, centrifuged at 3000 rpm for 5 min, followed by two rinses with Phosphate-Buffered Saline (PBS). Following this, 500 μL of binding buffer was slowly added, and the cells were then gently mixed to resuspend. Then, 5 μL of FITC reagent and 5 μL of PI fluorescent dye were introduced into the cell suspension. Flow cytometry (Thermo Scientific, USA) was used to assess the rate of apoptosis.

### Enzyme-linked immunosorbent assay

HUVECs cells were gently washed twice with PBS. Subsequently, 100 μL of Radioimmunoprecipitation Assay (RIPA) lysate was dispensed into each well, and the plates were placed on an ice bath and incubated for 10 min. The cells were subsequently spun at 12,000 rpm for 15 min at 4 °C to pellet cell debris, after which the supernatant was carefully aspirated. Malondialdehyde (MDA) Content Assay Kit (Solarbio, BC0025, Beijing), Superoxide Dismutase-1, Soluble (SOD1) Assay Kit (Beyotime, BSKH62259–96T, Shanghai), and Cellular Glutathione Peroxidase (GSH-Px) ELISA Kit (Beyotime, S0056, Shanghai) were used to measure the levels of oxidative stress indicators. Centrifuge the supernatant of HUVECs at 3000 rpm for 10 min, collect the pellet and polymer, and use a Human ceramide ELISA Kit (Hengyuan, B161268, Shanghai) to measure the level of C18 ceramide in the cells. Dual-luciferase reporter assay.

Bioinformatics databases were employed to predict downstream target genes of miR-1180–3p, and intersecting genes were filtered via Venn diagrams. GO functional enrichment analysis was performed on these intersecting genes. The direct interaction between miR-1180–3p and CERS1 was initially predicted via TargetScan 7.2, and subsequently validated using the dual-luciferase reporter assay.

Specifically, the binding sites between miR-1180–3p and CERS1 were inserted into the pGL3 vector to generate the wild-type CERS1 plasmid, while to generate the CERS1 mutant plasmid, the altered binding sites were cloned into the pGL3 vector. The constructed plasmids were co-transfected with miR-1180–3p mimics, miR-1180–3p inhibitors, or their corresponding negative controls into HUVECs cells. At 48 h after transfection, luciferase activity was measured to evaluate the target binding relationship between miR-1180–3p and CERS1.

### Statistical analysis

All data were subjected to statistical analysis and plotting using SPSS 26.0 and GraphPad Prism 9.0. For categorical variables, inter-group comparisons were analyzed using the Chi-Square test. For continuous variables, two-group comparisons were performed using the *t*-test, while multi-group comparisons were analyzed using one-way ANOVA, followed by Tukey’s multiple comparison test. Continuous variables are presented as mean ± SD for normally distributed variables; for non-normally distributed variables, they are presented as median (IQR), such as AHI. All cell experiments were performed with n = 3 biological replicates. Binary logistic regression was used to identify risk factors associated with the occurrence of OSAHS. The diagnostic value of miR-1180–3p was evaluated by plotting receiver ROC curves. Pearson correlation analysis was employed to assess the correlation between miR-1180–3p and the severity of OSAHS. A two-tailed p-value < 0.05 was deemed statistically significant.

The sample size was validated by a post-hoc power analysis using G*Power 3.1. With α = 0.05 (two-sided) and an expected effect size (*d* = 0.5), the achieved power reached 0.937 for the final cohort (112 OSAHS patients and 88 controls, n = 200), well above the 0.80 threshold, confirming sufficient statistical power.

## Results

### Study population baseline data

No significant differences were observed in baseline characteristics (including age, sex, BMI, hypertension history, diabetes history, LDL-C, HDL-C, TC, and TG) between all enrolled participants, demonstrating strong intergroup data comparability.

The OSAHS group demonstrated significantly altered sleep respiratory parameters compared to healthy controls. Versus healthy controls, the OSAHS group demonstrated significantly reduced LSpO_2_ alongside elevated AHI and TS90% values compared to controls (p < 0.001, Table 1).

**Table 1:** General information of the enrolled participants.

Parameters	Controls (n = 88)	OSAHS (n = 112)	p-value
Age (years)	51.85 ± 4.65	52.25 ± 4.71	0.552
Gender (Male, %)	45 (51.14)	59 (52.68)	0.828
BMI (kg/m^2^)	23.27 ± 2.83	23.98 ± 3.23	0.105
Hypertension (yes, %)	41 (46.59)	60 (53.57)	0.327
Diabetes mellitus (yes, %)	39 (44.32)	58 (51.79)	0.294
LDL-C (mmoL/L)	2.88 ± 0.33	2.93 ± 0.36	0.340
HDL-C (mmoL/L)	1.20 ± 0.39	1.12 ± 0.30	0.114
TC (mmoL/L)	4.25 ± 0.52	4.36 ± 0.68	0.222
TG (mmoL/L)	1.30 ± 0.21	1.33 ± 0.25	0.272
LSpO_2_ (%)	87.19 ± 7.24	72.18 ± 10.72	<0.001
AHI (events/h)	3.00 (1.00)	22.00 (11.00)	<0.001
TS90% (%)	0.95 ± 0.52	26.99 ± 8.60	<0.001

Notes: OSAHS, Obstructive Sleep Apnea Hypopnea Syndrome; BMI, Body Mass Index; LDL-C, Low-Density Lipoprotein Cholesterol; HDL-C, High-Density Lipoprotein Cholesterol; TC, Total Cholesterol; TG, Triglyceride; LSpO_2_, Lowest Oxygen Saturation by Pulse Oximetry; AHI, Apnea-Hypopnea Index; TS90%, Percentage of Sleep time when oxygen saturation is lower than 90%. Date was presented as mean ± SD, or n (%). p < 0.05 means a significant difference.

### miR-1180–3p expression level and its clinical diagnostic value

**Fig. 1:**
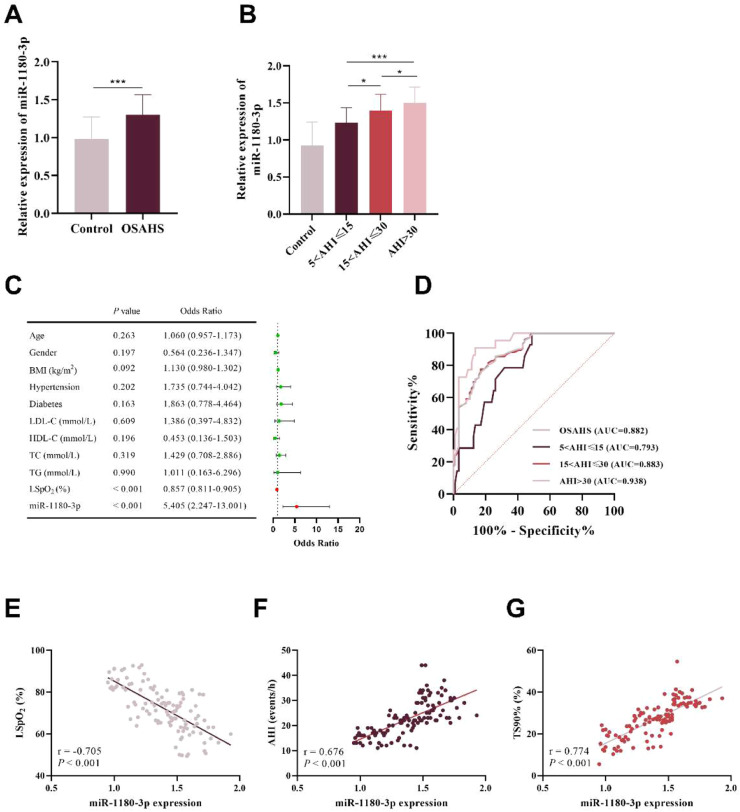
Aberrant miR-1180–3p expression levels and its diagnostic value in OSAHS. (A) miR-1180–3p exhibited significant upregulation in the serum of OSAHS patients. (B) miR-1180–3p levels in the severe group were significantly higher than those in the moderate and mild groups, and the levels in the moderate group were also significantly higher than those in the mild group. (C) miR-1180–3p was identified as an independent risk element in OSAHS progression. (D) The ROC curve demonstrated that miR-1180–3p exhibited favorable diagnostic performance for OSAHS and could effectively differentiate patients with mild, moderate, and severe disease. (E‒G) Pearson correlation assessment suggested that miR-1180–3p was markedly inversely related to LSpO_2_ (E) but demonstrated a strong direct correlation with AHI (F) and TS90% (G). *p < 0.05, ***p < 0.001.

RT-PCR quantification showed that serum miR-1180–3p expression was markedly upregulated in OSAHS patients compared to healthy controls (p < 0.001, Fig. 1A). Additionally, miR-1180–3p levels in patients in the severe group were significantly higher than those in the moderate (p = 0.047) and mild groups (p < 0.001), and the expression levels in the moderate group were significantly higher than those in the mild group (p = 0.013, Fig. 1B). Logistic regression analysis indicated that both miR-1180–3p (OR = 5.405, 95% CI 2.247‒13.001) and LSpO_2_ (OR=0.857, 95% CI 0.811‒0.905, Fig. 1C) were independent risk factors for OSAHS development. Specifically, elevated miR-1180–3p expression and reduced LSpO_2_ levels independently predicted higher OSAHS incidence. ROC analysis revealed that the diagnostic capability of miR-1180–3p in OSAHS (AUC = 0.882, 95% CI 0.836‒0.928) and effectively differentiated among mild (AUC = 0.793), moderate (AUC = 0.883), and severe OSAHS patients (AUC = 0.938, Fig. 1C).

Pearson correlation analysis was further performed to assess the association between miR-1180–3p and OSAHS severity. The data supported that miR-1180–3p inversely correlated with LSpO_2_ (*r* = −0.705, Fig. 1D), and directly correlated with AHI (*r* = 0.676, Fig. 1E) and TS90% (*r* = 0.774, Fig. 1F) in the study cohort.

**Fig. 2:**
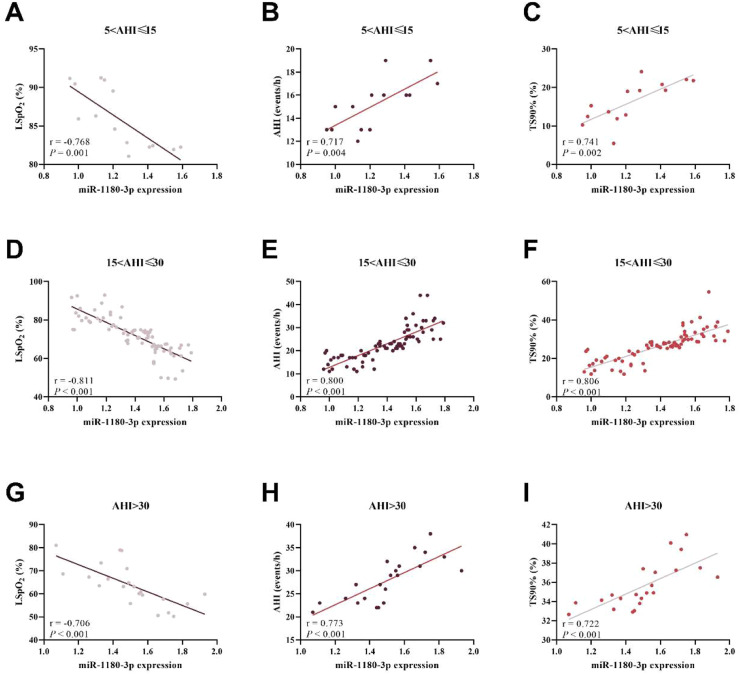
Correlation between the expression level of miR-1180–3p and clinical indicators of OSAHS in different subgroups. (A‒B) In the mild group, the expression of miR-1180–3p exhibited a significant negative correlation with LSpO_2_ (A) and significant positive correlations with AHI (B) and TA90% (C). (D‒F) In the moderate group, the expression of miR-1180–3p showed a significant negative correlation with LSpO_2_ (D) and significant positive correlations with AHI (E) and TA90% (F). (G‒I) In the severe group, the expression of miR-1180–3p showed a significant negative correlation with LSpO_2_ (G) and significant positive correlations with AHI (H) and TA90% (I).

Correlation analysis across subgroups showed that in patients with OSAHS of varying severities, miR-1180–3p exhibited significant correlations with core sleep indicators. In the mild group, the expression level of miR-1180–3p showed a significant negative correlation with LSpO_2_ (*r* = −0.768, Fig. 2A) and significant positive correlations with AHI (*r* = 0.717, Fig. 2B) and TS90% (*r* = 0.741, Fig. 2C). In the moderate group, this correlation remained significant: the expression of miR-1180–3p showed a significant negative correlation with LSpO_2_ (*r* = −0.811, Fig. 2D) and significant positive correlations with AHI (*r* = 0.800, Fig. 2E) and TS90% (*r* = 0.806, Fig. 2F). Similarly, in the severe group, these significant correlations remained: the expression of miR-1180–3p showed a significant negative correlation with LSpO_2_ (*r* = −0.706, Fig. 2G) and significant positive correlations with AHI (*r* = 0.773, Fig. 2H) and TA90% (*r* = 0.722, Fig. 2I).

### Regulatory effect of miR-1180–3p in CIH-induced HUVECs injury

**Fig. 3:**
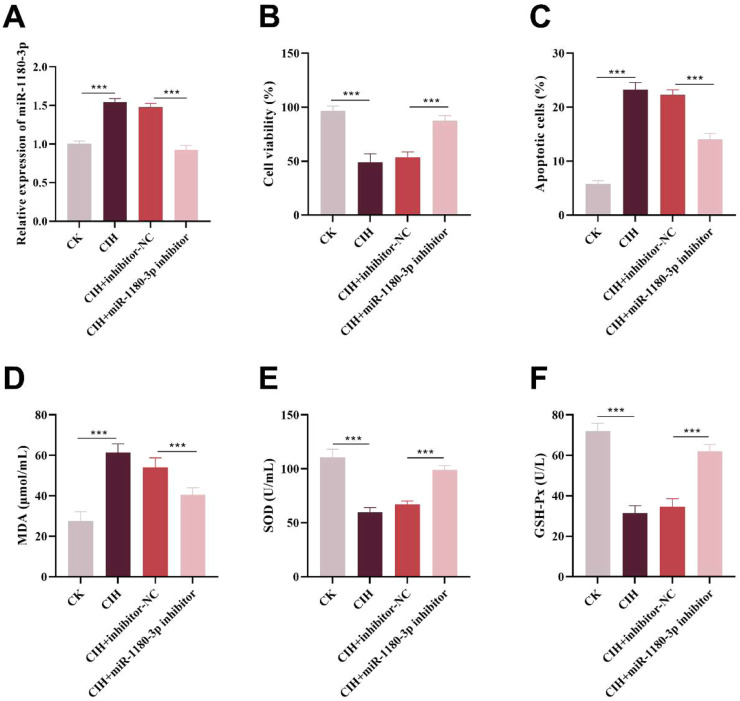
The involvement of miR-1180–3p in regulating CIH-induced HUVECs injury. (A) miR-1180–3p exhibited a marked increase in CIH-induced HUVECs. (B‒C) Reducing miR-1180–3p expression significantly enhanced cell viability (B) and reduced apoptosis (C) in CIH-induced HUVECs. (D‒F) miR-1180–3p inhibitor significantly reduced MDA levels (D) and elevated SOD (E) and GSH-Px levels (F). ***p < 0.001.

Compared with the control group, the expression level of miR-1180–3p in the CIH group was significantly upregulated, and its expression was markedly downregulated following transfection with a miR-1180–3p inhibitor (p < 0.001, Fig. 3A).

CCK-8 assay results showed that CIH treatment significantly reduced the viability of HUVECs compared with the control group, whereas knockdown of miR-1180–3p expression significantly restored cell viability (p < 0.001, Fig. 3B). Apoptosis assay results revealed that CIH treatment significantly increased the apoptosis rate of HUVECs, while downregulation of miR-1180–3p significantly reduced the apoptosis rate (p < 0.001, Fig. 3C).

Furthermore, ELISA assays further elucidated how miR-1180–3p modulates oxidative stress in HUVECs exposed to CIH. The results indicated that CIH treatment significantly elevated the level of MDA and reduced SOD and GSH-Px levels in HUVECs. Conversely, downregulation of miR-1180–3p reversed the CIH-induced injury in HUVECs, significantly inhibiting MDA level while increasing SOD and GSH-Px levels (p < 0.001, Fig. 3D‒F).

### Target binding between miR-1180–3p and CERS1

**Fig. 4:**
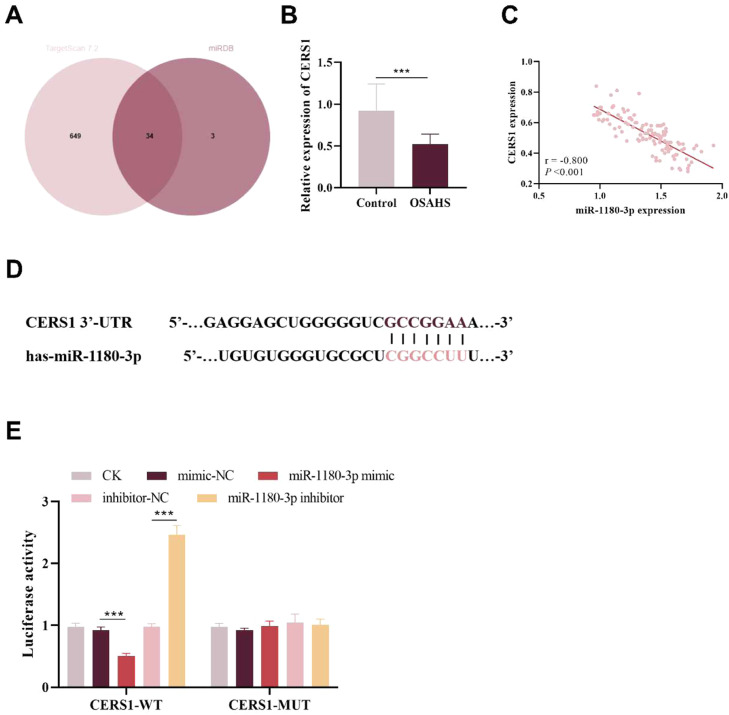
Validation of target binding between miR-1180–3p and CERS1. (A) 34 potential miR-1180–3p-regulated genes were identified through Venn diagram analysis. (B) CERS1 was significantly downregulated in the serum of OSAHS patients. (C) CERS1 expression was significantly negatively correlated with that of miR-1180–3p (D) Binding site between CERS1 and miR-1180–3p (E) The dual-luciferase reporter assay validated the target binding relationship between CERS1 and miR-1180–3p ***p < 0.001.

Downstream target genes of miR-1180–3p were predicted using the TargetScan7.2 and miRDB databases. The predictive results from the two databases were subsequently intersected and screened via a Venn diagram, ultimately leading to the identification of 34 common target genes (Fig. 4). A GO functional enrichment analysis was performed on 34 intersecting genes (Table S1). The results of GO enrichment analysis showed that among the top 20 significantly enriched biological processes, CERS1 was mainly involved in 8, and 6 of these pathways were directly related to mitochondrial autophagy and cellular REDOX homeostasis, such as mitophagy (GO:0,000423), regulation of autophagy of mitochondrion (GO:1,903146), regulation of mitochondrion organization (GO:0,010821), positive regulation of mitochondrion organization (GO:0,010822), autophagy of mitochondrion (GO:0,000422), mitochondrion disassembly (GO:0,061726). This result suggested that the abnormal expression of CERS1 can affect the normal physiological function of mitochondria, and the absence of CERS1 may disrupt the dynamic balance of mitochondria. While the function and structure of mitochondria is intimately linked to the pathophysiological processes of OSAHS, with CIH-induced mitochondrial dysfunction driving ROS accumulation and endothelial apoptosis. Additionally, RT-PCR results revealed that serum CERS1 expression was significantly downregulated in OSAHS patients (p < 0.001, Fig. 4). A strong negative correlation was observed between CERS1 expression and miR-1180–3p (*r* = −0.800, Fig. 4C). Therefore, CERS1 was chosen as the target gene for further study.

TargetScan 7.2 predicted miR-1180–3p binding motifs in CERS1 (Fig. 4D). The targeting binding relationship was validated using a dual-luciferase reporter assay. The results demonstrated that miR-1180–3p mimics significantly inhibited the luciferase activity of the CERS1-WT construct, whereas miR-1180–3p inhibitors significantly enhanced this activity (p < 0.001, Fig. 4E), indicating the presence of a direct targeting binding relationship between miR-1180–3p and CERS1.

### Mechanism of miR-1180–3p targeting CERS1 to regulate CIH-induced HUVECs injury

**Fig. 5:**
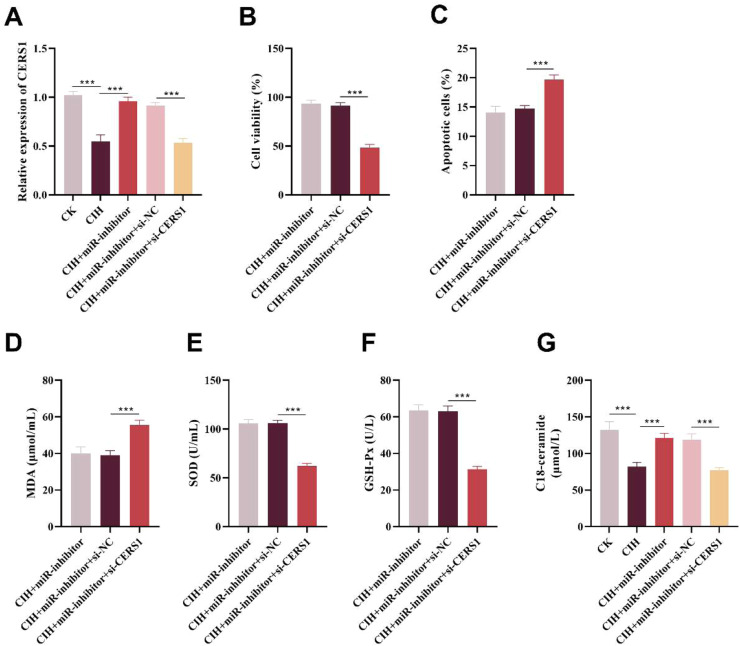
miR-1180–3p targeted CERS1 to regulate CIH-induced injury of HUVECs. (A) Inhibition of miR-1180–3p resulted in significantly increased CERS1 levels, while si-CERS1 led to a decrease in its expression. (B‒C) CERS1 knockdown abolished miR-1180–3p-mediated protection against CIH-induced injury of HUVECs, such as inhibiting cell viability (B) and promoting apoptosis (C). (D‒F) si-CERS1 reversed the protective effect of miR-1180–3p inhibitor on CIH-induced HUVEC injury by significantly upregulating MDA level (D) and downregulating SOD (E) and GSH-Px levels (F). (G) CIH treatment significantly reduced C18 ceramide levels, whereas miR inhibitors effectively reversed this inhibitory effect; after silencing CERS1, C18 ceramide levels decreased significantly again. ***p < 0.001.

In comparison with non-treated experimental controls, the expression level of CERS1 in HUVECs was significantly reduced following CIH treatment. On this basis, transfection with a miR-1180–3p inhibitor significantly upregulated CERS1 expression; however, silencing CERS1 via siRNA markedly downregulated its expression (p < 0.001, Fig. 5A).

Furthermore, CERS1 knockdown significantly attenuated the protective effect of the miR-1180–3p inhibitor against CIH-induced HUVECs injury, leading to reduced cell viability and promoted cell apoptosis (p < 0.001, Fig. 5B‒C).

ELISA results demonstrated that CERS1 silencing significantly elevated the level of MDA while decreasing SOD and GSH-Px levels (p < 0.001, Fig. 5D‒F). Collectively, these findings indicate that miR-1180–3p regulates the progression of OSAHS by targeting CERS1. Additionally, the authors measured the levels of C18 ceramide in cells from each group using an ELISA assay. The results revealed that CIH induction significantly reduced C18 ceramide levels, whereas the miR-1180–3p inhibitor markedly increased C18 ceramide levels (p < 0.001). Notably, silencing CERS1 also significantly suppressed C18 ceramide levels (p < 0.001, Fig. 5G). Overall, the trend of changes in C18 ceramide levels was consistent with the trend of changes in CERS1 expression levels.

## Discussion

OSAHS is a prevalent disorder characterized by recurrent episodes of upper airway collapse during sleep, leading to Chronic Intermittent Hypoxia (CIH), sleep fragmentation, and increased cardiovascular risk.^1^ The pathological link between OSAHS and cardiovascular complications, such as hypertension and heart failure, is strongly associated with CIH-induced endothelial dysfunction. While the Apnea-Hypopnea Index (AHI) remains a diagnostic cornerstone, its limitations in capturing disease burden and cardiovascular risk highlight the need for novel biomarkers that reflect underlying pathophysiological processes.^17^ Emerging evidence implicates dysregulated miRNAs and bioactive lipids in mediating hypoxia-related cellular damage.^6^^,^^7^ This study identifies a novel miR-1180–3p/CERS1 axis and elucidates its role in OSAHS pathogenesis, offering new insights into the molecular mechanisms linking CIH to vascular injury. miR-1180–3p has been shown to exhibit good diagnostic and predictive value in various diseases,^18^^,^^19^ which aligns with the findings of this study. Furthermore, the ROC curve further validated that miR-1180–3p possesses favorable diagnostic efficacy for OSAHS. It is crucial to contextualize its value within the broader field through comparative analysis. Through a systematic literature review, the authors found that the diagnostic AUC values of reported circulating miRNAs associated with OSAHS exhibit a relatively wide spectrum. In a previous study, Yang et al. reported several miRNAs with diagnostic potential, whose AUC values were miR-145–5p (0.849), miR-126–3p (0.822), let-7d-5p (0.778), miR-320b (0.751), miR-107 (0.733), and miR-26a-5p (0.729).^20^ Additionally, recent studies have reported higher AUC values, such as miR-149–3p (0.876)^6^ and miR-107 (0.946).^21^ The AUC of miR-1180–3p places it within the competitive upper range of this spectrum, supporting its potential as a diagnostic biomarker. More importantly, the present study moves beyond mere diagnostic association. The authors found that miR-1180–3p levels were significantly correlated with key polysomnographic indices of disease severity, including AHI, LSpO₂, and TS90%. Crucially, subgroup analysis confirmed that these correlations persisted within distinct severity strata (mild, moderate, and severe OSAHS). This indicates that miR-1180–3p is not merely a marker of disease presence, but exhibits a continuous, graded relationship with disease severity. This finding is critical, as it suggests that the upregulation of miR-1180–3p is not merely a dichotomous marker for the presence or absence of OSAHS, but rather a potential internal biomarker that changes continuously and progressively with disease severity.

A key finding of this study is the identification of CERS1 as a direct functional target of miR-1180–3p CERS1, a member of the ceramide synthase family, regulates ceramide synthesis and is widely involved in various cellular processes.^22^ Previous studies have reported the function of CERS1 in cancer cell proliferation and apoptosis,^23^ along with its potential in cancer diagnosis and prognosis.^24^ Currently, research on the pathological mechanisms of OSAHS has advanced to explore aspects including oxidative stress, chronic inflammation, and apoptosis. As a “pro-apoptotic lipid”, ceramide accumulation is closely associated with oxidative stress, endoplasmic reticulum stress, and mitochondrial dysfunction.^25^ Wu X et al. demonstrated that hypoxia activates mitophagy, thereby triggering a series of oxidative stress responses,^26^ which is consistent with the results of GO functional enrichment analysis in this study. Furthermore, previous studies have indicated that miR-574–5p can regulate the alternative splicing process of CERS1 by synergizing with HDAC1, leading to dysregulation of the ceramide profile and thereby promoting disease progression.^27^

C18-ceramide is a specific subtype within the ceramide family, and CERS1 can directly regulate its generation. Wang et al. reported that overexpression of CERS1 significantly upregulates C18-ceramide levels,^28^ which is consistent with the present findings. Previous studies have shown that C18-ceramide accelerates the apoptosis of cancer cells and exhibits significantly low expression in cancer tissues.^29^ In the present study, after CIH treatment, the levels of C18-ceramide in HUVECs decreased significantly, suggesting that CIH may reduce C18-ceramide synthesis by inhibiting CERS1 expression or interfering with its enzymatic activity, thereby weakening the cell’s adaptability to damage. The observed changes in C18-ceramide levels associated with apoptotic phenotypes in this study may involve several highly conserved downstream signaling pathways. First, as a critical pro-apoptotic lipid second messenger, C18-ceramide can induce Mitochondrial Outer Membrane Permeabilization (MOMP), promoting the release of cytochrome c into the cytosol. This event activates initiator Caspase-9 and subsequently the effector Caspase-3, ultimately executing the apoptosis program, a pathway well-documented in various cellular models.^30^^,^^31^ Second, ceramide can precisely modulate the balance of Bcl-2 family proteins. For instance, it inhibits the activity of the anti-apoptotic member Bcl-2 while facilitating the mitochondrial translocation and oligomerization of the pro-apoptotic protein Bax, thereby governing the initiation of the mitochondrial apoptotic pathway.^32^ Furthermore, the crucial survival signaling pathway PI3K/AKT is also a potential target. Ceramide acts as a potent activator of Protein Phosphatase-2A (PP2A), which can mediate the dephosphorylation and inactivation of AKT. This, in turn, relieves AKT's suppression of downstream pro-apoptotic factors (e.g., Bad, FoxO) and attenuates its mediated cell survival signals, thereby cooperatively promoting apoptosis.^28^ Together, these literature-supported mechanisms form a coherent framework explaining how C18-ceramide may converge on mitochondrial function and survival signaling hubs to regulate cellular fate decisions.

In this study, through dual-luciferase reporter assays, the authors validated the target binding relationship between miR-1180–3p and CERS1. The identification of miR-1180–3p as a novel regulator of CERS1 expands the understanding of the molecular mechanisms underlying hypoxia-induced endothelial dysfunction, a key driver of cardiovascular diseases. Further co-inhibition experiments revealed that co-inhibiting miR-1180–3p and CERS1 exacerbated CIH-induced HUVEC injury, suggesting that miR-1180–3p exacerbates CIH-induced vascular endothelial damage by targeting CERS1, thereby promoting the pathological progression of OSAHS.

However, this study has several limitations. First, retrospective design and sample limitations. Due to the retrospective nature based on historical samples, the remaining volume after miR-1180–3p detection was insufficient for high-quality experimental validation and direct comparison with other candidate miRNAs. In addition, the single-center design of this study may limit the generalizability of the findings. Future prospective, multi-center cohort studies will be designed to simultaneously detect and compare multiple candidate miRNAs (e.g., miR-126, miR-149–3p) from the outset. Second, the study population of this research comprises OSAHS patients diagnosed based on indicators such as AHI and TS90%. This study design limits the clinical interpretability of the AUC values of indicators like AHI and TS90% in the current cohort. Furthermore, although baseline characteristics (age, gender, BMI) showed no significant differences between groups, confounding effects from other unmeasured factors cannot be entirely ruled out. In future studies, the authors plan to expand the cohort to include a more diverse population ‒ including OSAHS patients with comorbidities, individuals with borderline AHI values, OSAHS patients with atypical symptoms, and healthy controls with sleep-related symptoms but no elevated AHI ‒ to more comprehensively validate the clinical generalizability of the relevant indicators. Furthermore, when evaluating the association between miR-1180–3p and OSAHS risk, the OR initially reported in this study was solely based on univariate logistic regression analysis. Although univariate analysis showed no significant correlation for common covariates such as age, sex, and BMI, the failure to perform multivariable adjustment to further control potential confounding effects constitutes a limitation in the statistical methodology of this study. Future studies should plan and conduct more comprehensive multivariable model adjustment at the design stage. Third, this study primarily focuses on the correlation between miR-1180–3p and endothelial dysfunction in OSAHS patients, but the specific molecular pathways through which CERS1 regulates apoptosis (e.g., mitochondrial pathways, PI3K/AKT pathways) were only indirectly speculated. Future studies need to further validate the effects of CERS1 silencing on cleaved caspase-3, Bcl-2/Bax, and AKT phosphorylation using techniques such as Western blot to complete the mechanistic chain. Fourth, lack of experimental validation for the downstream pathways of C18-ceramide. Constrained by experimental conditions, this study did not directly assess the activation status of key downstream apoptotic and survival molecules of C18-ceramide, such as Caspase-3, Bcl-2/Bax, and PI3K/AKT. To bridge this gap in the mechanistic cascade, subsequent research will employ molecular biology techniques like Western blotting to quantitatively analyze the expression and activation levels of these critical signaling molecules. This will be combined with functional rescue experiments using specific pathway inhibitors or agonists to confirm the precise pathways through which C18-ceramide acts within the present model.

## Conclusion

In summary, the expression of miR-1180–3p in the serum of OSAHS patients was significantly upregulated and exhibited good clinical diagnostic value. miR-1180–3p is proposed as a potential biomarker for OSAHS. By targeting CERS1, miR-1180–3p exacerbated CIH-induced damage to HUVECs, thereby contributing to the pathological progression of OSAHS.

## Data availability statement

Data must be requested from the corresponding author.

## Ethics approval and consent to participate

Approval was obtained from the ethics committee of Fuzhou Hospital of Traditional Chinese Medicine Affiliated to Fujian University of Traditional Chinese Medicine (No. 2021126, on 11 September 2021). The procedures used in this study adhere to the tenets of the Declaration of Helsinki. Written informed consent was obtained from all individual participants included in the study.

## Consent for publication

Consent for publication was obtained for every individual person’s data included in the study.

## Authors’ contributions

Conceptualization, M.D., Y.C., Q.C.; Data curation, M.D., Y.C., H.C., Q.C.; Formal analysis, M.D., Y.C., H.C., Q.C.; Funding acquisition, M.D., Y.C.; Investigation, M.D., Y.C., H.C., Q.C.; Methodology, M.D., Y.C., H.C.; Project administration, Q.C.; Resources, M.D., Y.C., H.C., Q.C.; Software, M.D., Y.C., H.C., Q.C.; Supervision, Q.C.; Validation, M.D., Y.C., H.C., Q.C.; Visualization, M.D., Y.C., H.C.; Roles/Writing-original draft, M.D., Y.C.; Writing-review & editing, Q.C.

## Funding

This study was funded by Fujian Provincial Key Clinical Specialty Construction Project (No. 20230601); Fuzhou Hospital of Traditional Chinese Medicine Science and Technology Program (No. 2024-Ynky-02); Clinical Special Project of the University-Level Research Program, Fujian University of Traditional Chinese Medicine（No. XB2025089）.

## Declaration of competing interest

The authors declare no conflicts of interest.

## Appendix Supplementary materials

Image, application 1

## Footnotes

Supplementary material associated with this article can be found, in the online version, at doi:10.1016/j.clinsp.2026.100944.
