# Supplementary material for: miR-1180–3p as a biomarker of obstructive sleep apnea hypopnea syndrome and its role in chronic intermittent hypoxia-induced vascular injury
Source: Clinics (Sao Paulo). 2026 Apr 23;81:100944. doi: 10.1016/j.clinsp.2026.100944 (PMC13127203; doi:10.1016/j.clinsp.2026.100944)
Supplement: Supplementary file 1 [file mmc1.docx]

**CLINICS-D-25-01321**

**Table S1.** Top 20 Significantly Enriched Biological Process (BP) Terms in GO Analysis

| **ID** | **Description** | **pvalue** | **geneID** | **Count** | **Enrichment.Score** | **Fold.Enrichment** |
| --- | --- | --- | --- | --- | --- | --- |
| GO:0070301 | cellular response to hydrogen peroxide | 0.00056196 | ETS1/FXN/PINK1 | 3 | 3.250294836 | 18.48880843 |
| GO:0000423 | mitophagy | 0.000976322 | CERS1/PINK1 | 2 | 3.010406875 | 43.140553 |
| GO:1903146 | regulation of autophagy of mitochondrion | 0.001356742 | CERS1/PINK1 | 2 | 2.867502762 | 36.60410557 |
| GO:0010821 | regulation of mitochondrion organization | 0.001710367 | CERS1/FXN/PINK1 | 3 | 2.766910679 | 12.58266129 |
| GO:0042542 | response to hydrogen peroxide | 0.001779162 | ETS1/FXN/PINK1 | 3 | 2.749784387 | 12.41029607 |
| GO:0034614 | cellular response to reactive oxygen species | 0.00211014 | ETS1/FXN/PINK1 | 3 | 2.67568883 | 11.68969823 |
| GO:0090199 | regulation of release of cytochrome c from mitochondria | 0.002854069 | FXN/PINK1 | 2 | 2.544535502 | 25.16532258 |
| GO:0010823 | negative regulation of mitochondrion organization | 0.002972459 | FXN/PINK1 | 2 | 2.526884147 | 24.65174457 |
| GO:0002931 | response to ischemia | 0.004139629 | CSF1/PINK1 | 2 | 2.383038571 | 20.82647386 |
| GO:2000649 | regulation of sodium ion transmembrane transporter activity | 0.004139629 | FXYD5/HECW1 | 2 | 2.383038571 | 20.82647386 |
| GO:0001836 | release of cytochrome c from mitochondria | 0.004280477 | FXN/PINK1 | 2 | 2.368507871 | 20.47348278 |
| GO:0040014 | regulation of multicellular organism growth | 0.004568782 | FXN/CSF1 | 2 | 2.340199579 | 19.80222105 |
| GO:0016239 | positive regulation of macroautophagy | 0.004865853 | CERS1/PINK1 | 2 | 2.312841048 | 19.17357911 |
| GO:0061912 | selective autophagy | 0.005646503 | CERS1/PINK1 | 2 | 2.248220405 | 17.76375712 |
| GO:1902305 | regulation of sodium ion transmembrane transport | 0.005646503 | FXYD5/HECW1 | 2 | 2.248220405 | 17.76375712 |
| GO:0000302 | response to reactive oxygen species | 0.005787451 | ETS1/FXN/PINK1 | 3 | 2.237512701 | 8.161726242 |
| GO:0010822 | positive regulation of mitochondrion organization | 0.006653878 | CERS1/PINK1 | 2 | 2.176925173 | 16.32345248 |
| GO:0000422 | autophagy of mitochondrion | 0.007924568 | CERS1/PINK1 | 2 | 2.101024426 | 14.91278375 |
| GO:0061726 | mitochondrion disassembly | 0.007924568 | CERS1/PINK1 | 2 | 2.101024426 | 14.91278375 |
| GO:0034765 | regulation of ion transmembrane transport | 0.008388489 | FXYD5/KCNA6/PINK1/HECW1 | 4 | 2.076316242 | 4.92030747 |
